# Epidemiology of Astigmatism in Japan: Analysis of More Than 9,000,000 Spectacle Prescriptions

**DOI:** 10.1167/iovs.67.4.67

**Published:** 2026-04-28

**Authors:** Shizuka Koh, Hidehito Matsuoka, Kiyotaka Hori, Ryo Kawasaki, Kohji Nishida

**Affiliations:** 1 Department of Innovative Visual Science, The University of Osaka Graduate School of Medicine, Suita, Osaka, Japan; 2 Department of Ophthalmology, The University of Osaka Graduate School of Medicine, Suita, Osaka, Japan; 3 Medical Strategy Department, JINS Inc., Chiyoda-ku, Tokyo, Japan; 4 Public Health, Department of Social Medicine, The University of Osaka Graduate School of Medicine, Suita, Osaka, Japan

**Keywords:** astigmatism, refractive error, large-scale data, spectacle wearer, epidemiology

## Abstract

**Purpose:**

The purpose of this study was to characterize the prevalence of astigmatism and stratify it by age, sex, and geographic region and evaluate interocular differences in cylinder power in a large-scale Japanese population using spectacle prescription data.

**Methods:**

This retrospective cross-sectional study analyzed de-identified spectacle prescription data collected from a nationwide optical retail chain between September 2021 and August 2023. The targeted individuals were aged 6 to 89 years. They were categorized based on their cylinder power and axis orientation. Step 1 involved evaluating these parameters across different ages, sexes, and regions. Step 2 involved quantifying the interocular differences in the individuals with astigmatism (cylinder power of ≥0.25 diopter [D]) in at least one eye.

**Results:**

We analyzed 9,204,993 prescriptions (50.8% female subjects). The cylinder power increased progressively with age, and this was accompanied by a shift from with-the-rule (WTR) to against-the-rule (ATR) orientation. Men had slightly higher cylinder powers and a higher prevalence of ATR astigmatism than women. The regional variation across Japan was modest. Interocular comparison revealed that 81.6% and 9.4% of the individuals had differences in cylinder power of ≤0.50 D and ≥1.00 D, respectively. These findings indicate substantial bilateral symmetry in most individuals.

**Conclusions:**

This nationwide analysis of spectacle prescription data provides an updated epidemiologic overview of astigmatism in Japan. We characterized the age-, sex-, and region-related variations in cylinder power and axis orientation and quantified the interocular asymmetry at the population level. These findings highlight the value of large-scale prescription databases as a novel resource for population-based vision research.

Astigmatism is one of the most common refractive errors, characterized by unequal refractive power across different meridians of the eye, resulting in blurred or distorted vision. From a clinical and epidemiological perspective, astigmatism is often described as refractive astigmatism (the total astigmatic error measured at the spectacle plane) and corneal astigmatism, with the former reflecting the net contribution of corneal and internal optical components. In this context, refractive astigmatism is particularly relevant because it directly determines visual performance and the need for optical correction. It has long been recognized as an important determinant of visual performance. It influences optical image quality and neural aspects of visual development when uncorrected during early life.^1^^–^^3^ The clinical relevance of astigmatism extends from infancy, when it may be associated with amblyopia, to adulthood, when it affects optical quality and visual satisfaction after cataract or refractive surgery. The widespread adoption of toric intraocular and contact lenses has made it increasingly important to understand the distribution and severity of astigmatism in the general population to optimize visual correction strategies and address the population burden of astigmatism.^4^ Uncorrected or inadequately corrected astigmatism degrades visual acuity, contrast sensitivity, and functional visual performance.^5^^,^^6^ Furthermore, astigmatism may adversely affect visually demanding daily tasks, including night driving and digital device use.^7^^–^^9^ Appropriate astigmatic correction, particularly with toric lenses, improves visual performance and reduces visual discomfort compared to spherical correction.^8^^–^^10^

Research and public interest in myopia control have increased recently.^11^ However, astigmatism has received less attention despite its substantial impact on visual function. Large-scale epidemiological studies and systematic reviews from diverse populations worldwide^4^^,^^12^ have consistently demonstrated that astigmatism is one of the most prevalent refractive errors, with reported prevalence varying widely depending on age, ethnicity, and diagnostic criteria. Its prevalence has been reported to range from 8% to 62% in East Asia and is approximately 40% globally.^4^ Age-related changes in the lenticular and corneal components contribute to the increasing prevalence and gradual shift from with-the-rule (WTR) to against-the-rule (ATR) astigmatism.^2^^,^^13^^–^^16^ However, the findings on sex and regional differences have been inconsistent.^4^

Previous epidemiological studies and systematic reviews from diverse populations have consistently reported age-related shifts in astigmatism magnitude and axis orientation across the lifespan.^4^ Several population-based studies in Japan have also provided valuable insights.^17^^–^^19^ However, most have focused on middle-aged and older adults. Therefore, younger age groups have been under-represented,^17^^–^^19^ despite the effects of mild to moderate astigmatism on visual comfort and optical quality in younger individuals.^20^^,^^21^ Therefore, understanding its epidemiological distribution across a wide age range is essential. Spectacle prescription records, which are the primary source of refractive correction data, offer a practical and large-scale means of exploring population-level refractive characteristics. In contrast with traditional epidemiologic studies that are often constrained by sample size or geography, retail prescription data capture the characteristics of a vast, demographically diverse population and have emerged as a valuable “big data” resource for public health and vision science.

The present study analyzed spectacle prescription data from a nationwide optical retail chain in Japan to characterize the distribution of astigmatism by age, sex, and geographic region, and evaluate the interocular differences in cylinder power in a large population. Given the unprecedented scale of this real-world dataset, the study was designed as a large-scale descriptive epidemiological analysis to characterize demographic and regional patterns of astigmatism in a nationwide population. In this study, astigmatism specifically refers to refractive astigmatism quantified using spectacle prescription cylinder power.

## Methods

This retrospective, observational study was approved by the Institutional Review Board of The University of Osaka Hospital (registration number: 24231(T1)) and conducted in accordance with the tenets of the Declaration of Helsinki, as revised in 2013. An opt-out method was used to obtain individual consent. All data were anonymized prior to analysis.

Spectacle prescription data from September 2021 to August 2023 were collected from a large optical retail chain (JINS Inc., Maebashi, Japan) operating 434 stores nationwide at the time of the study. The dataset included the de-identified electronic records of the customers who purchased spectacles and underwent refraction measurements during this period. Only prescriptions issued by ophthalmologists were accepted and used for the customers aged 15 years or younger. The electronic database recorded the final spectacle prescriptions at the time of purchase. Their spherical and cylindrical components were extracted and analyzed. These prescriptions included both external prescriptions issued by ophthalmologists and prescriptions determined through in-store refraction in optical retail settings. The astigmatism analyzed in this study represents refractive astigmatism based on spectacle prescription cylinder power, and does not correspond to corneal astigmatism measured by keratometry or corneal topography. In optical retail settings in Japan, refraction measurements are primarily obtained using autorefractors as objective measurements, and the final spectacle prescriptions are determined based on these measurements in routine commercial practice. Individuals who were wearing contact lenses prior to refraction were measured after lens removal according to routine practice, typically after an interval of at least 10 minutes. It should be noted that spectacle prescriptions reflect prescribed refractive correction in real-world clinical and commercial settings and may not necessarily represent the true physiological refractive status measured under standardized clinical conditions.

### Analytical Flow and Parameters

#### Study Population

**Figure 1.**
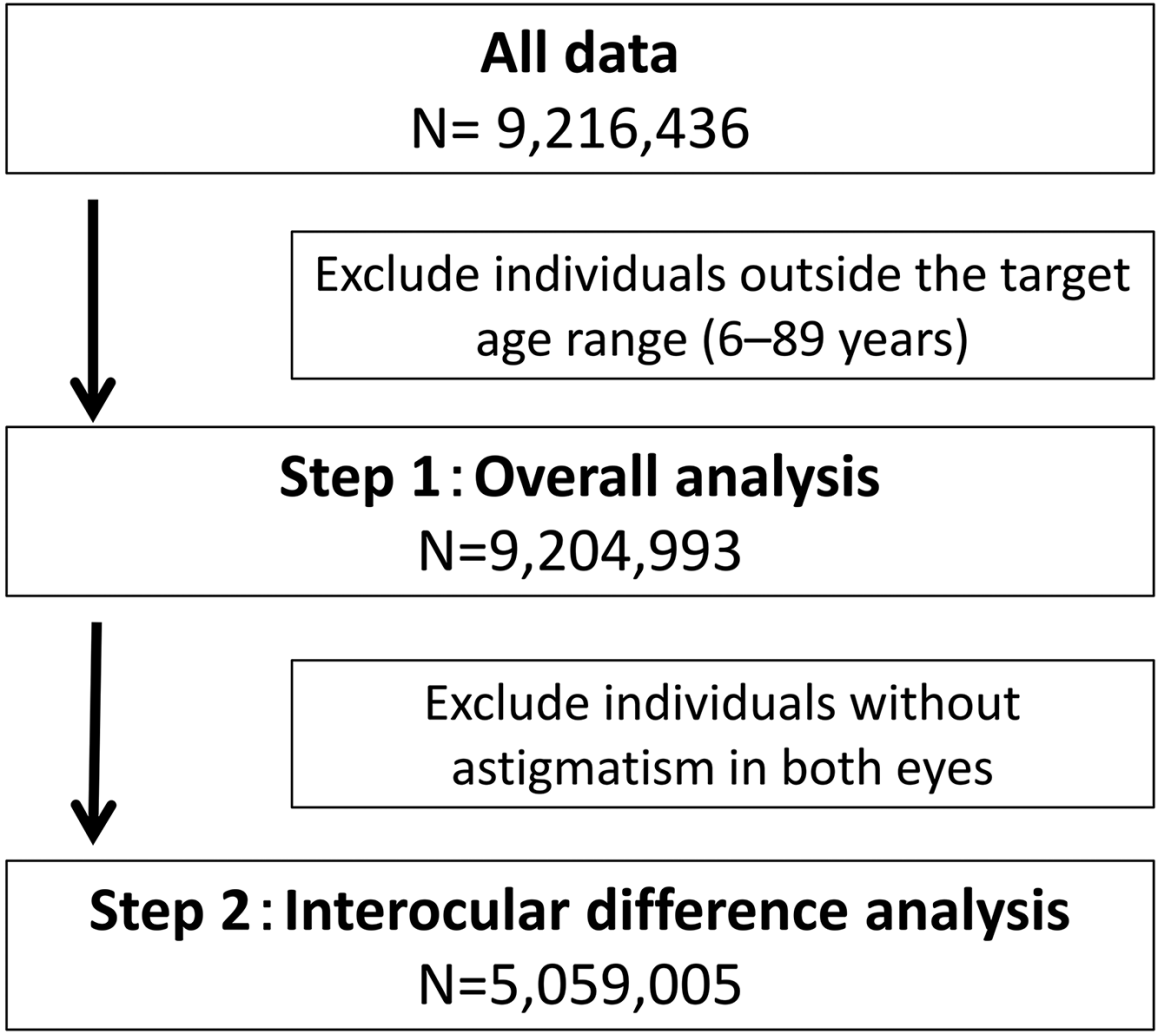
**Flowchart of the study population selection.** A total of 9,216,436 records were initially screened. Individuals outside the target age range (6–89 years) were excluded, leaving the data of 9,204,993 individuals for the overall analysis (step 1). Of these, the data of 5,059,005 individuals with ≥0.25 D of astigmatism in at least one eye were included in the interocular difference analysis (step 2).

A total of 9,216,436 records were initially extracted from the database. After excluding individuals outside the predefined age range (6–89 years), 9,204,993 individuals were included in the overall analysis. For the interocular difference analysis, individuals with astigmatism (cylinder power ≥0.25 diopter [D] in at least one eye) were further selected, resulting in a final sample of 5,059,005 individuals. The flow of study population selection is shown in Figure 1.

#### Step 1: Overall Analysis

As the analysis was restricted to individuals aged 6 to 89 years, those outside this range were excluded. Individuals younger than 6 years were excluded because reliable and standardized refraction is difficult to obtain in routine retail settings in this age group, and spectacle prescriptions in very young children are often issued for amblyopia management rather than routine refractive correction. In addition, the upper age limit of 89 years was defined based on the available sample size in the database to ensure sufficient statistical stability in each age stratum. Therefore, the analytical age range was set at 6 to 89 years. The distributions and characteristics of astigmatism in the population were determined based on the analysis of all eligible prescriptions. The degree (cylinder power) and type (axis orientation) of astigmatism were evaluated according to age, sex, and geographic region.

#### Step 2: Interocular Difference Analysis

The individuals with no astigmatism in both eyes were excluded. Those with astigmatism of ≥0.25 D in at least one eye were included. The interocular difference in the cylinder power was determined to characterize the astigmatic asymmetry.

Astigmatism was categorized based on the cylinder power as follows: none (0 D), minimal (0 D < and < 0.75 D), mild (0.75 D ≤ and < 1.5 D), moderate (1.5 D ≤ and < 2.5 D), and severe (2.5 D ≤). The astigmatic axis was classified into WTR (axis between 0 and 30 degrees or 150 and 180 degrees), ATR (axis between 60 degrees and 120 degrees), and oblique (all remaining axes, i.e., 31–59 degrees and 121–149 degrees). These classification criteria were based on commonly used definitions in previous epidemiological studies of astigmatism, which have used similar thresholds for cylinder power and axis orientation.

### Statistical Analyses

The demographic and refractive characteristics of the study population were described using counts and percentages. The primary aim of this study was to provide an overview of the prevalence and distribution of astigmatism. Comparisons between groups (e.g., age, sex, or geographic region) were performed to aid interpretation and contextualization of the findings.

Chi-squared tests were used for analyses involving categorical variables, including comparisons of axis type distributions. Independent samples *t*-tests were used to assess differences in mean cylinder power between the two groups (e.g., by sex). Age-related trends in mean cylinder power were evaluated using one-way analysis of variance, and post hoc Tukey tests were used to determine the pairwise differences between age groups. Age-related trends in the proportions of astigmatism axis types were evaluated using the Cochran–Armitage trend test. In addition, to evaluate whether regional differences could be explained by differences in age and sex, we performed multivariable regression analyses adjusting for these factors. For continuous outcomes (e.g., cylinder power), multiple linear regression analyses were conducted with region, age group, and sex included as independent variables. For categorical outcomes (e.g., astigmatism type), multinomial logistic regression analyses were performed with the same set of covariates. Given the very large sample size of this study, even trivial differences could reach statistical significance. Therefore, we focused on effect size-based measures rather than relying solely on *P* values. Specifically, we calculated standardized regression coefficients (β), partial coefficients of determination (partial *R*²) for linear models, and partial pseudo-coefficients of determination (pseudo-*R*²) for logistic models, and permutation importance to evaluate the relative contribution of each explanatory variable to the outcomes in a complementary manner. The correlations between the right and left cylinder powers were examined using Pearson's correlation coefficients. All statistical analyses were performed using the DuckDB, Matplotlib, SciPy, Tableone, NumPy, and Pandas packages for Python (version 3.12; The Python Software Foundation, USA). Statistical significance was set at *P* < 0.05.

## Results

A total of 9,204,993 individuals were included in the overall analysis. The study population consisted of 50.8% female subjects and 49.2% male subjects, with a mean age of 36.5 ± 16.3 years (range = 6–89 years). Sample size and demographic distribution of participants by geographic region, age group, and sex are shown in Supplementary Table S1.

### Step 1: Overall Analysis

#### Age and Cylinder Power

**Figure 2.**
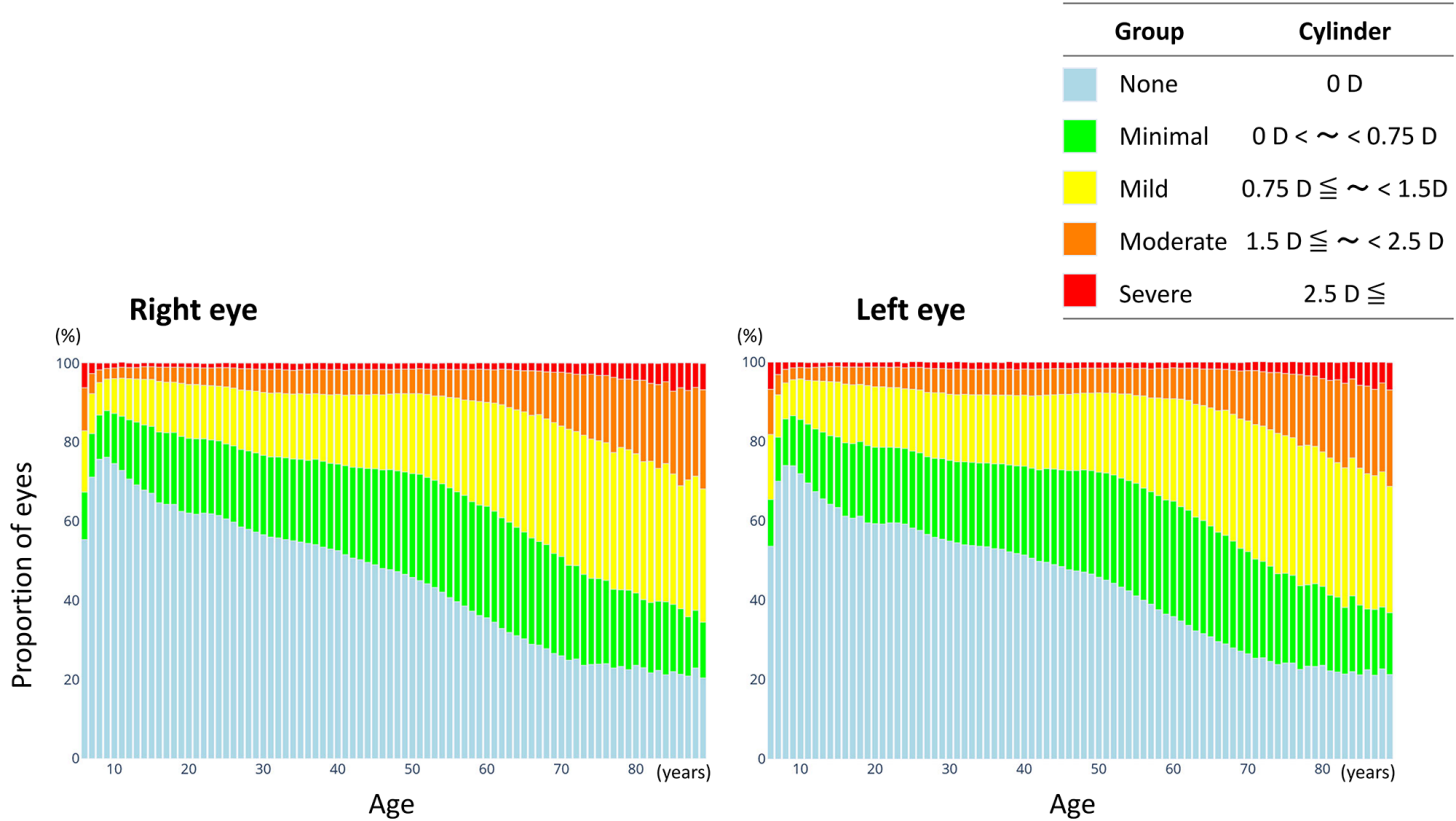
**Cylinder powers for the right and left eyes stratified by age group.** The stacked bar charts show the proportions of eyes in each astigmatism category (none, minimal, mild, moderate, and severe) across the age groups. The prevalence and magnitude of astigmatism increased progressively with age, with a marked increase in mild-to-severe astigmatism after approximately 50 years of age.

 Figure 2 stratifies the cylinder power by age group for both eyes. The prevalence and severity of astigmatism increased progressively with age. The proportion of individuals without astigmatism (0 D) decreased after early adulthood, whereas those with mild, moderate, and severe astigmatism increased markedly after approximately 50 years of age. Table 1 stratifies the mean cylinder power for the right and left eyes by age group. The mean cylinder power increased with age (ANOVA with post hoc Tukey test, *P* < 0.001).

**Table 1.** Cylinder Powers for the Right and Left Eyes Stratified by Age Group

Age Group	Cylinder Power, Right Eye	Cylinder Power, Left Eye
6–9 y	0.26 ± 0.59 D	0.28 ± 0.61 D
10–19 y	0.27 ± 0.52 D	0.30 ± 0.54 D
20–29 y	0.33 ± 0.57 D	0.36 ± 0.59 D
30–39 y	0.39 ± 0.62 D	0.41 ± 0.63 D
40–49 y	0.43 ± 0.61 D	0.43 ± 0.62 D
50–59 y	0.48 ± 0.61 D	0.47 ± 0.60 D
60–69 y	0.61 ± 0.63 D	0.59 ± 0.62 D
70–79 y	0.78 ± 0.70 D	0.76 ± 0.68 D
80–89 y	0.93 ± 0.79 D	0.91 ± 0.78 D

D, diopter.

Statistical analysis: Differences across age groups were evaluated using 1-way ANOVA with post hoc Tukey test (*P* < 0.001 for both eyes).

#### Age-Related Distribution of the Astigmatic Axis

**Figure 3.**
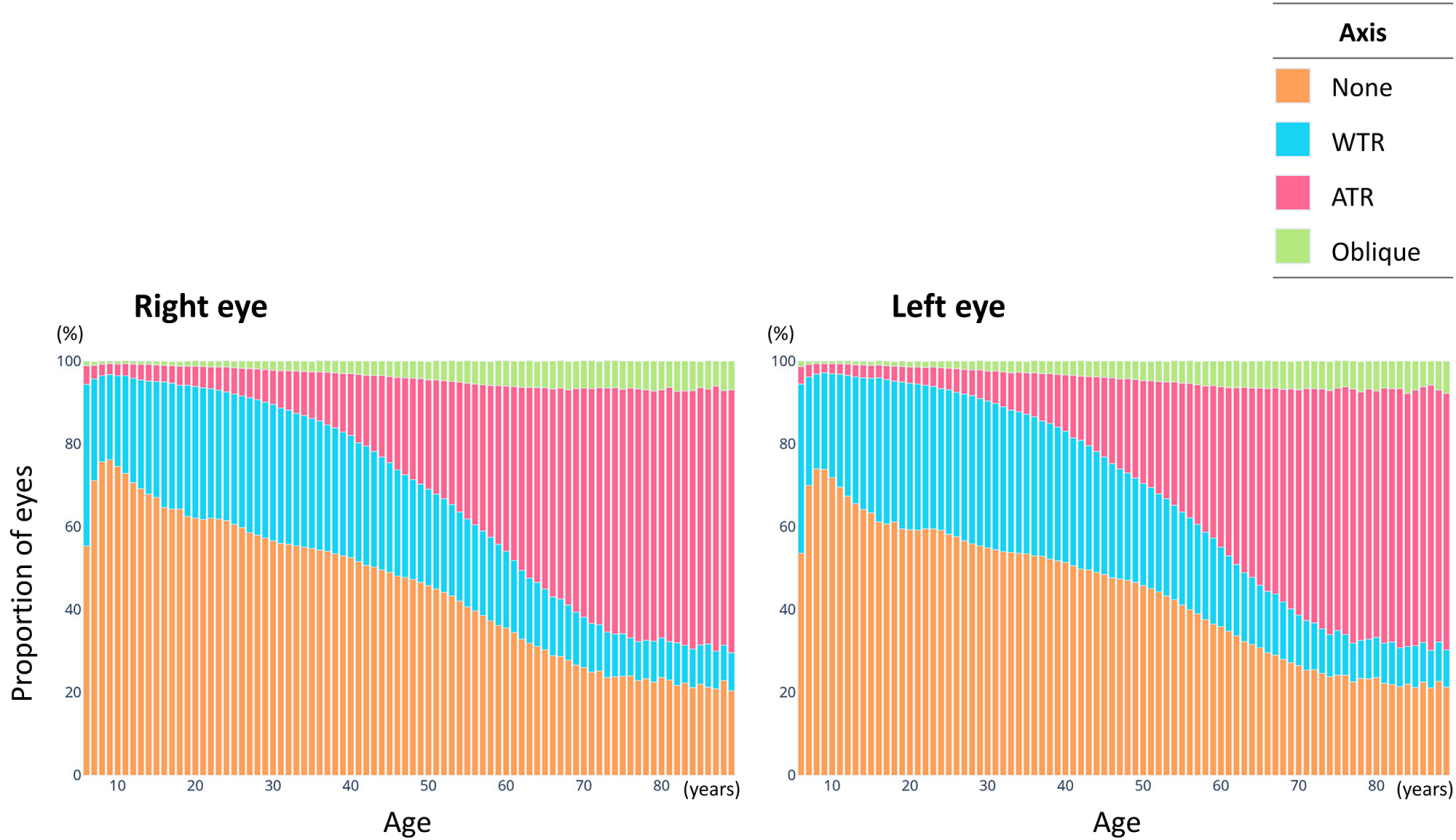
**Age-related distribution of astigmatic axis types for the right and left eyes.** The proportions of eyes with with-the-rule (WTR), against-the-rule (ATR), oblique, and non-astigmatism are provided for the different age groups. WTR astigmatism was predominant in the younger age groups, but its prevalence decreased steadily with age. In contrast, the prevalence of ATR astigmatism increased progressively with age. The prevalence of oblique astigmatism increased until approximately 60 years of age and stabilized thereafter.

**Figure 4.**
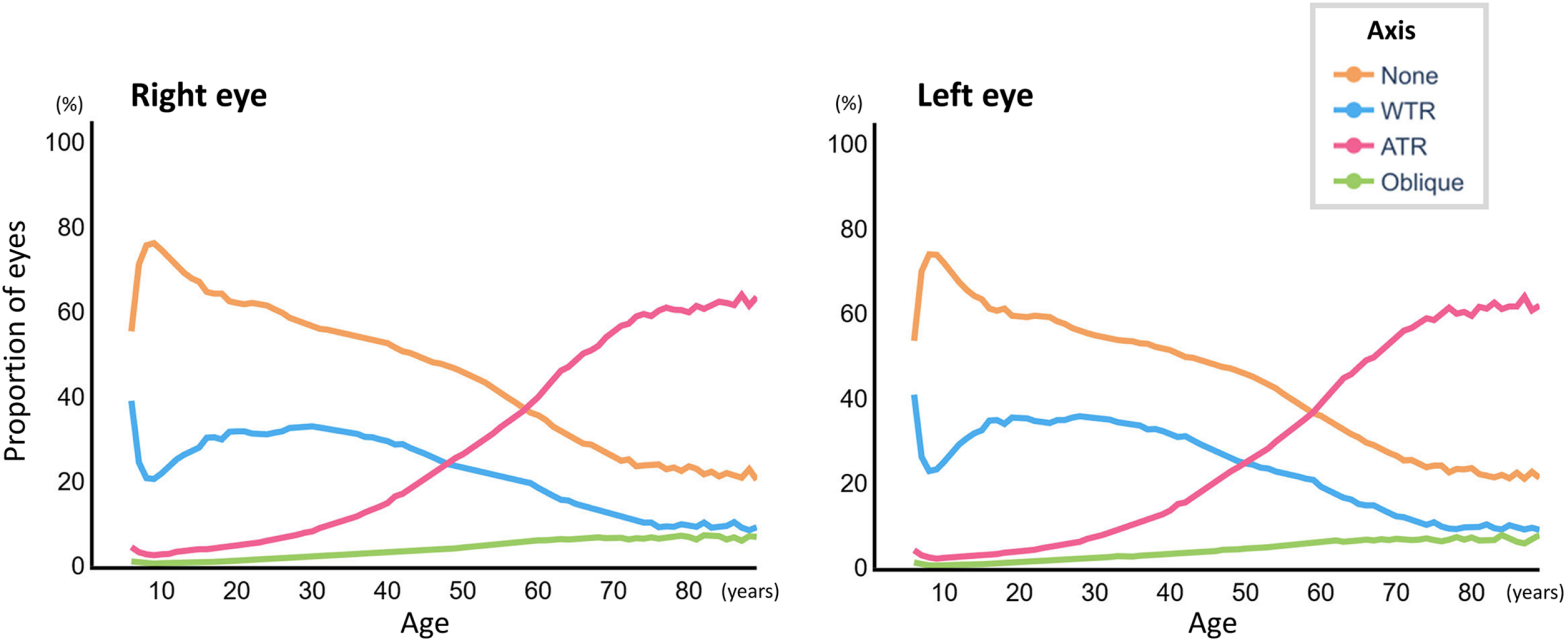
**Age-related trends in the proportions of astigmatic axis types for the right and left eyes.** The line graphs illustrate the age-specific proportions of with-the-rule (WTR) and against-the-rule (ATR) astigmatism in the right and left eyes. Consistent with the Cochran–Armitage trend test, the proportion of WTR astigmatism showed a significant monotonic decrease with age (*P* < 0.001), whereas the proportion of ATR astigmatism showed a significant monotonic increase with age (*P* < 0.001).

 Figure 3 shows the distribution of axis types by age group for both eyes. WTR astigmatism was predominant in the younger individuals but its prevalence decreased steadily with age. In contrast, the prevalence of ATR astigmatism increased progressively with age. The prevalence of oblique astigmatism increased until approximately 60 years of age and remained nearly constant. The Cochran–Armitage trend test revealed a significant age-related monotonic decrease in the proportion of WTR astigmatism (*P* < 0.001), with a peak in early adulthood followed by a gradual decline with aging. In contrast, the proportion of ATR astigmatism showed a significant monotonic increase with age (*P* < 0.001; Fig. 4).

#### Sex Differences

**Figure 5.**
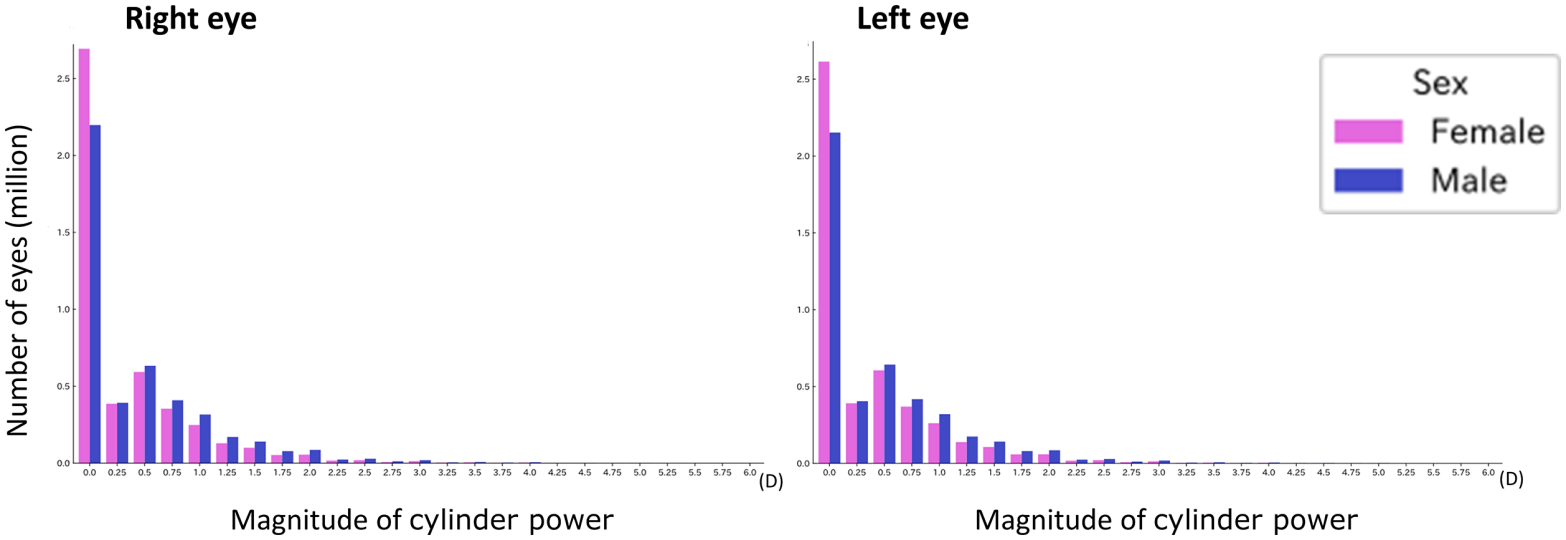
**Sex-related differences in cylinder power.** The histograms of the cylinder power for the male subjects and female subjects are provided. The mean cylinder power for the males was slightly higher after excluding the data of the individuals without astigmatism in both eyes.

 Figure 5 compares the cylinder power distributions for male and female patients. The mean cylinder powers for the right and left eyes were slightly higher for male patients than for female patients (−0.76 ± 0.67 D vs. −0.68 ± 0.62 D and −0.78 ± 0.67 D vs. −0.72 ± 0.63 D, respectively; *P* < 0.001, *t*-test) after excluding the individuals without astigmatism in both eyes. Women had a higher proportion of cases without astigmatism and smaller cylinder magnitudes. The distributions of the astigmatic axes for the right and left eyes of the female and male groups were significantly different (*P* < 0.001, chi-squared test; Table 2). The proportions of WTR, ATR, and oblique astigmatism for the right eye were 25.5%, 14.3%, and 2.6%, respectively, for female subjects and 28.2%, 19.8%, and 3.4%, respectively, for male subjects. The corresponding proportions for the left eye were 28.4%, 13.1%, and 2.7%, respectively, for female subjects and 30.2%, 18.7%, and 3.6%, respectively, for male subjects.

**Table 2.** Distribution of the Astigmatic Axes for the Right and Left Eyes of the Female and Male Groups

Astigmatic Axis	None	WTR	ATR	Oblique
Right eye				
F	57.6%	25.5%	14.3%	2.6%
M	48.5%	28.2%	19.8%	3.4%
Left eye				
F	55.9%	28.4%	13.1%	2.7%
M	47.5%	30.2%	18.7%	3.6%

ATR, against-the-rule astigmatism; WTR, with-the-rule astigmatism.

Statistical analysis: The distributions of astigmatic axis between female patients and male patients were compared using the chi-squared test (*P* < 0.001 for both right and left eyes).

#### Regional Differences

The regional variations in the degree and type of astigmatism across the nine geographic areas of Japan were generally modest but noticeable. They were Hokkaido, Tohoku, Kanto, Chubu, Kansai, Chugoku, Shikoku, Kyushu, and Okinawa. Detailed data are provided in the Supplementary Analysis (see Supplementary Fig. S1). In the multivariable analyses, age showed the largest contribution to the outcomes, followed by sex, whereas the contribution of region was relatively small. Detailed results, including standardized regression coefficients, partial *R*², and permutation importance, are presented in Supplementary Table S2. These findings suggest that the observed regional differences are largely explained by differences in age and sex composition rather than reflecting substantial intrinsic regional variation. In addition, the imbalance in sample size across regions may also have contributed to the apparent regional differences.

### Step 2: Interocular Difference Analysis

The interocular difference analysis included 5,059,005 individuals (46.6% female subjects and 53.4% male subjects) with astigmatism (≥0.25 D) in at least one eye. Their mean age was 39.9 ± 16.4 years.

**Figure 6.**
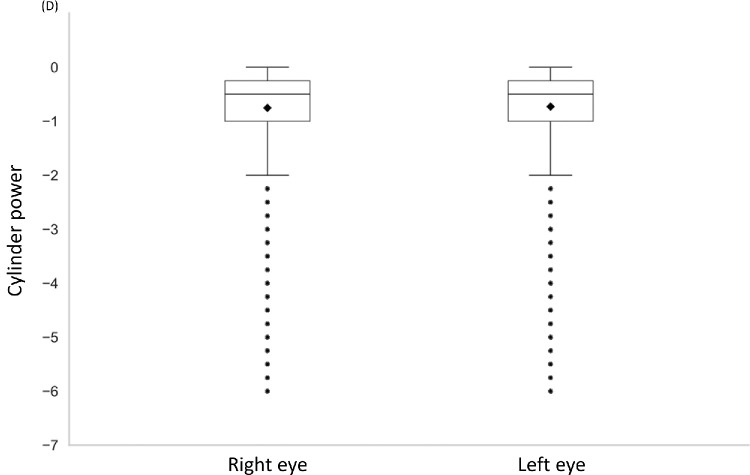
**Distribution of cylinder power for the right and left eyes of individuals with astigmatism.** The box-and-whisker plots depict the distribution of cylinder power for each eye.

**Figure 7.**
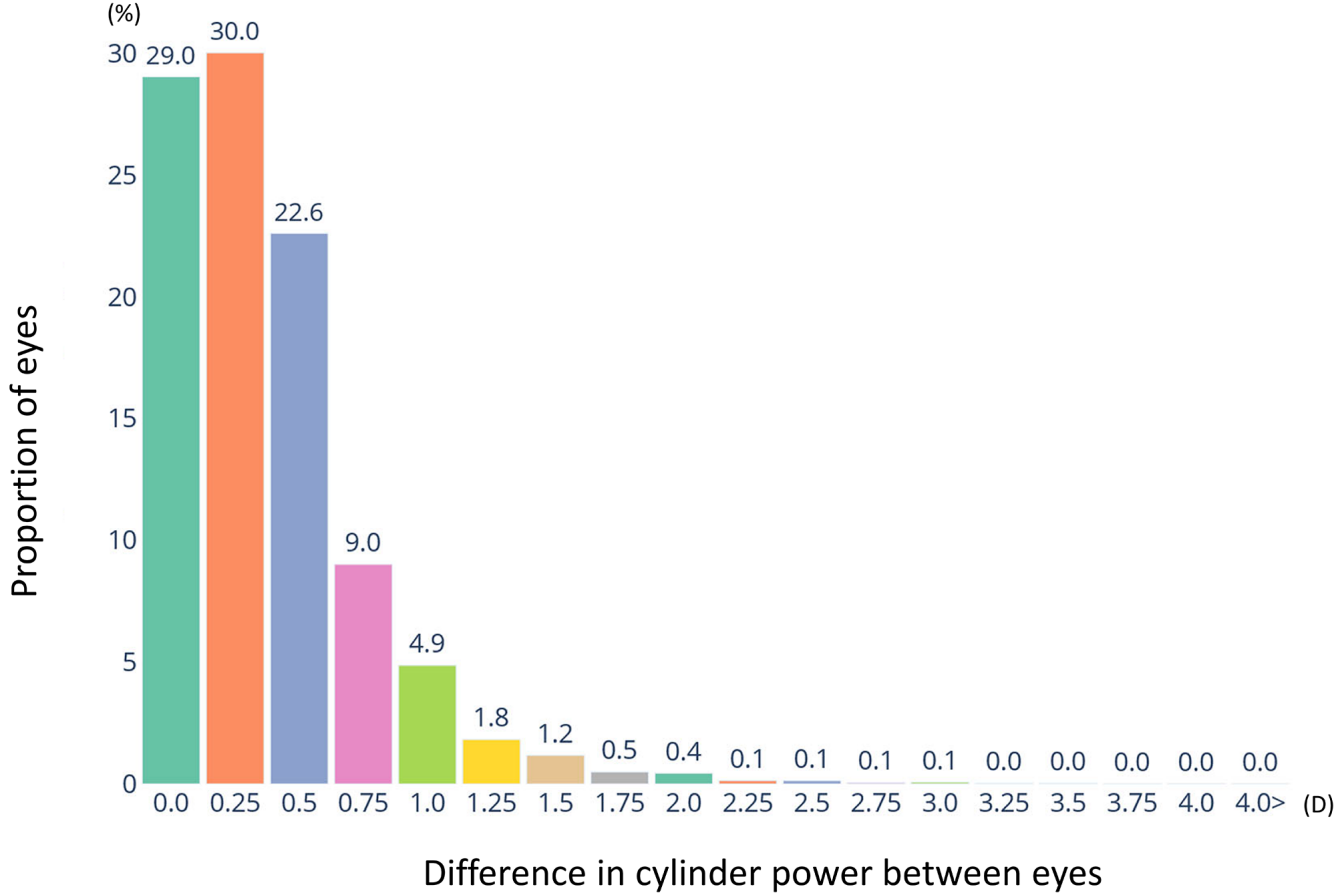
**Distribution of interocular differences in cylinder power.** Histogram showing the absolute difference in cylinder power between the eyes of the individuals with astigmatism (cylinder power, ≥0.25 D). The proportions of individuals with identical cylinder powers, those with differences of ≤0.50 D, and those with differences of ≥1.00 D were 29.0%, 81.6%, and 9.4%, respectively.

The distributions of the astigmatic prescriptions for both eyes are shown in the box and whisker plots (Fig. 6). The mean cylinder powers for the right and left eyes were −0.72 ± 0.65 D and −0.75 ± 0.65 D, respectively. A significant positive correlation was observed between the right and left eye cylinder powers (*r* = 0.644, *P* < 0.001; Pearson correlation), indicating that the severity of astigmatism was similar for both eyes. The distributions of the interocular differences in the cylinder powers of the individuals with astigmatism are shown in Figure 7. The cylinder powers of 29.0% of the individuals were identical for both eyes, indicating no interocular difference. The absolute interocular differences in the cylinder powers of 81.6% and 9.4% of the individuals were ≤0.50 D and ≥1.00 D, respectively. These findings indicate that most individuals had comparable severity of astigmatism for both eyes, and only a few had marked asymmetry.

## Discussion

Myopia control has been the focus of research in recent years. However, the present findings highlight the continued importance of understanding astigmatism to maintain optimal visual performance. This study provides the most comprehensive epidemiologic overview of astigmatism in the Japanese population to date based on more than nine million spectacle prescriptions collected across Japan. Although the present analysis provides a large-scale epidemiologic overview, it should be interpreted as an analysis of prescribed astigmatism rather than the true biological prevalence of astigmatism.

These age-related shifts in astigmatism axis and magnitude are generally consistent with trends reported in epidemiological studies from various populations,^4^^–^^12^ suggesting that the observed patterns may reflect fundamental age-related changes in ocular and refractive characteristics rather than population-specific phenomena. Three major epidemiologic studies^17^^–^^19^ have provided foundational data on the prevalence and characteristics of astigmatism in Japan. Asano et al.^17^ reported the effect of aging on astigmatism based on the data of 2161 individuals aged 40 to 79 years from 2 small Japanese communities. They demonstrated a clear age-related increase in cylinder power, which established the basis for understanding age-associated refractive changes. Sawada et al.^18^ reported the detailed distribution of the astigmatic axis based on the data of 2829 adults from a large-scale epidemiologic investigation of glaucoma prevalence. They confirmed the gradual shift from WTR to ATR astigmatism with aging but found no significant sex difference in the magnitude of cylinder power. Namba et al.^19^ provided direct evidence of the progressive increase in lenticular astigmatism with age and its contribution to the age-related ATR shift in the Japanese population based on the findings of a longitudinal study involving the same cohort for 10 years. The present nationwide analysis of more than 9 million spectacle prescriptions for individuals aged 6 to 89 years confirmed these age-related trends. The cylinder power and the proportion of ATR astigmatism increased gradually with age, which is consistent with previous reports.^4^ However, our dataset covers a wider age range that captures both pediatric and older adults, and it provides a more comprehensive overview of age-dependent refractive changes than previous studies that relied on subjective refraction data from limited sample sizes. Our analysis revealed statistically significant but modest differences in cylinder power and axis distribution. Men had slightly higher mean cylinder powers, whereas women had a higher proportion of non-astigmatic prescriptions. This contrasts with the findings of Sawada et al.,^18^ who reported no notable sex difference in refractive astigmatism in a Japanese adult population. The discrepancy may reflect methodological differences and the substantially larger sample size in our study, which likely enabled the detection of subtle sex-related trends that smaller cohorts could not identify.

Previous large-scale studies conducted outside Japan have frequently reported a higher prevalence of ATR astigmatism in men.^22^^–^^24^ For example, men had significantly higher odds of having ATR astigmatism based on data from the National Health and Nutrition Examination Survey.^22^ Studies from northern England and Israel have also reported earlier onset of ATR changes in men.^23^^,^^24^ However, these findings are not universal; for example, a population-based study from Spain reported no significant association between sex and axis orientation.^25^ Our cohort had a higher proportion of men with ATR astigmatism, which is consistent with the findings of several previous studies. Women had a higher proportion of non-astigmatic prescriptions and lower prevalence of WTR astigmatism; WTR astigmatism was slightly more frequent in men. These collective observations suggest that the overall axis distribution may vary with sex depending on demographic, racial, and methodological contexts. Recent Japanese population studies have also documented sex-related differences in ocular structure and refractive characteristics, including anterior segment biometry, refractive error, and higher-order aberrations in children.^26^^,^^27^ These findings support the plausibility of sex-related variation in astigmatic axis orientation in our dataset. Our results indicate that sex-related differences in astigmatic axis distribution exist in the Japanese population, although the specific proportions differ from those reported in other regions. The biological mechanisms underlying these differences remain uncertain, but sex-related variations in eyelid tension, corneal structural properties may play a role. In addition, sex hormones have been suggested to influence corneal biomechanics and curvature through their effects on collagen organization and corneal hydration, which could indirectly affect astigmatic axis orientation.^22^ However, direct evidence linking hormonal factors to population-level astigmatism patterns remains limited. Further studies are needed to clarify how these anatomic and hormonal factors interact to shape the observed differences.

Regional differences in the distribution of astigmatism were observed, but they were relatively modest. Small variations across Japan were observed, but they should be interpreted with caution because the number of retail stores contributing data was not evenly distributed across regions; urban areas were more heavily represented. In addition, regional differences in age distribution are likely to exist in this large retail dataset. Because astigmatism showed a strong age-dependent pattern in our analysis, the observed regional variations may partly reflect differences in age structure rather than true geographic or environmental effects. The regional differences observed in this study should be interpreted in the context of differences in population composition. As shown in Supplementary Table S1, the distribution of participants varied across regions, with approximately 40% of the sample concentrated in the Kanto region. In our analysis, both the magnitude and axis distribution of astigmatism were strongly associated with age, suggesting that differences in age distribution across regions may have substantially influenced the observed regional patterns. Furthermore, the multivariable regression analyses demonstrated that age had the largest contribution to the outcomes, followed by sex, whereas the contribution of region was relatively small (see Supplementary Table S2). Taken together, these findings indicate that a substantial proportion of the observed regional differences may be explained by differences in demographic composition, particularly age and sex, rather than reflecting intrinsic geographic variation. These findings highlight the importance of accounting for demographic structure when interpreting geographic variations in large-scale observational data. However, the use of data from a nationwide optical retail chain provides a unique advantage by enabling the detection of subtle geographic patterns that would be difficult to capture in smaller population-based studies. Such large-scale, continuously collected datasets could serve as a valuable complement to traditional epidemiologic surveys and support future analyses of environmental or lifestyle factors that may influence refractive characteristics at the population level.

The interocular difference analysis revealed that 81.6% of the individuals in our cohort had a difference in cylinder power of ≤0.50 D between their eyes, indicating a high degree of bilateral refractive symmetry. This finding aligns with those of Luensmann et al.,^28^ who analyzed the data of more than 80,000 patients with astigmatism from optometrist practices in the United States, the United Kingdom, and Canada and reported that 82.1% had an interocular difference of ≤0.50 D. The similarity in distribution patterns across distinct populations suggests that interocular balance in astigmatism is a robust and consistent feature of human refractive development. The difference in mean cylinder power reported by Luensmann et al.^28^ was slightly higher than that observed in our dataset. However, this discrepancy is likely attributable to differences in the study population and clinical setting. Their cohort was obtained from optometric clinics in Western countries, whereas our data were collected from a large optical retail chain in Japan and encompassed a broader and more heterogeneous general population. These demographic, racial, and service-context differences may contribute to small variations in refractive magnitude but are unlikely to affect the overall interocular symmetry pattern. Both studies reported strong positive correlations between the cylinder powers of the two eyes (*r* = 0.64 for our dataset and *r* = 0.72 for the study by Luensmann et al.^28^). This supports the reliability and biological plausibility of this association across diverse populations. Approximately 9% of our individuals had an interocular difference of ≥1.00 D, which is comparable to the 10% reported by Luensmann et al.^28^ This represents a clinically relevant level of asymmetry that may influence binocular vision, spectacle adaptation, or toric contact lens fitting. Given the extremely large sample size, even very small interocular differences can reach statistical significance. However, interocular differences below approximately 0.50 D are generally considered clinically negligible in routine refractive practice. Therefore, the high proportion of individuals with ≤0.50 D interocular difference in our dataset suggests clinically meaningful bilateral symmetry despite the statistical detectability of minor variations.

Recent studies from various regions, including Western countries (primarily in North America),^29^ and Korea,^30^ have consistently reported a mismatch between the prevalence of clinically significant astigmatism and the frequency of its appropriate correction. Luensmann et al.^29^ reported that nearly half of the spectacle prescriptions they reviewed involved clinically relevant astigmatism (≥0.75 D). They also reported that approximately 80% of these prescriptions could be corrected using available ranges of soft toric lenses. However, the actual clinical utilization of toric lenses was substantially lower, highlighting the persistent gap between the proportions of patients who could benefit from toric correction and those who actually receive it. Chu et al.^30^ reported that fewer than 20% of spectacle wearers with astigmatism (≥0.75 D) in Korea had been fitted with toric lenses and more than half were undercorrected when they switched to contact lenses. Our current Japanese dataset complements these findings from an epidemiologic perspective. The proportions of cases of mild (0.75–1.50 D) and moderate (1.50–2.50 D) astigmatism exceeded 20% by early adulthood and increased progressively thereafter. Our analysis did not include contact lens prescriptions. However, these trends suggest that accurate detection and timely correction of low-to-moderate astigmatism, especially in younger adults, may help reduce long-term undercorrection. Large-scale spectacle prescription data^28^^–^^30^ can provide valuable insights for understanding population-level refractive needs and informing future public health and educational strategies related to visual correction.

This study has several limitations. First, the spectacle prescription data were obtained from individuals who visited optical retail stores for vision correction and therefore represent a selected, help-seeking real-world population rather than a population-based sample. This introduces potential ascertainment bias and means that the findings should not be interpreted as reflecting the true prevalence of astigmatism in the general population, with possible under-representation of individuals without astigmatism. The dataset was large and included data from stores nationwide. However, it was not exhaustive and may not fully represent the entire Japanese population. The estimates for regions with smaller sample sizes may be less reliable, and urban areas were relatively over-represented. Second, the analysis was based on the final spectacle prescriptions rather than direct clinical measurements. In addition, refraction measurements were obtained across multiple optical retail stores and external medical institutions. Although most retail stores used similar commercial autorefractor devices, some variability in instruments and measurement environments may have existed. Furthermore, prescriptions for individuals aged 15 years or younger were based on clinical assessments from different medical institutions, where devices and measurement protocols were not fully standardized. Therefore, our findings should be interpreted as reflecting real-world prescribing patterns and refractive correction needs rather than exact physiological refractive error. Spectacle prescriptions are influenced not only by objective refractive measurements but also by subjective refraction, prescribing habits, visual demands, and tolerance to astigmatic correction, which may result in systematic differences from manifest or cycloplegic refraction. Third, the dataset lacked clinical information, such as visual acuity and ocular history. Individuals with refractive findings suggestive of ocular disease were typically advised to seek ophthalmologic examination. However, information on ocular histories, including surgical status and existing eye diseases, was not excluded and was therefore not fully accounted for. Fourth, the interocular analyses were based on single-visit data and did not consider measurement variability. Finally, the cross-sectional design precludes longitudinal evaluation. In addition, the data were collected between 2021 and 2023, during the coronavirus disease 2019 (COVID-19) pandemic. Because pre-pandemic prescription data were not available in this database and the analysis was cross-sectional, the potential impact of pandemic-related lifestyle changes (e.g., increased near work and reduced outdoor activity) on astigmatism distribution could not be directly evaluated.

Future studies involving the linkage of retail prescription databases with clinical and biometric data or longitudinal epidemiologic and school screening cohorts are warranted to address these limitations and provide a more comprehensive understanding of the natural history of astigmatism. These integrated approaches could establish a sustainable framework for nationwide refractive surveillance and guide more effective public health and educational strategies related to visual correction.

## Conclusions

This nationwide analysis of more than nine million spectacle prescriptions provides the most comprehensive characterization of the distribution of astigmatism in Japan to date. The results highlight consistent age-related and interocular trends, modest sex and regional differences, and a high prevalence of mild-to-moderate astigmatism even in younger adults. These findings underscore the continuing importance of accurate detection and appropriate correction of astigmatism to ensure optimal visual performance and quality of life across the population.

## Supplementary Material

Supplement 1

Supplement 2

Supplement 3
