# Supplementary material for: Epidemiology of Astigmatism in Japan: Analysis of More Than 9,000,000 Spectacle Prescriptions
Source: Invest Ophthalmol Vis Sci. 2026 Apr 28;67(4):67. doi: 10.1167/iovs.67.4.67 (PMC13150933; doi:10.1167/iovs.67.4.67)
Supplement: Supplement 1 [file iovs-67-4-67_s001.pdf]

Fig S1

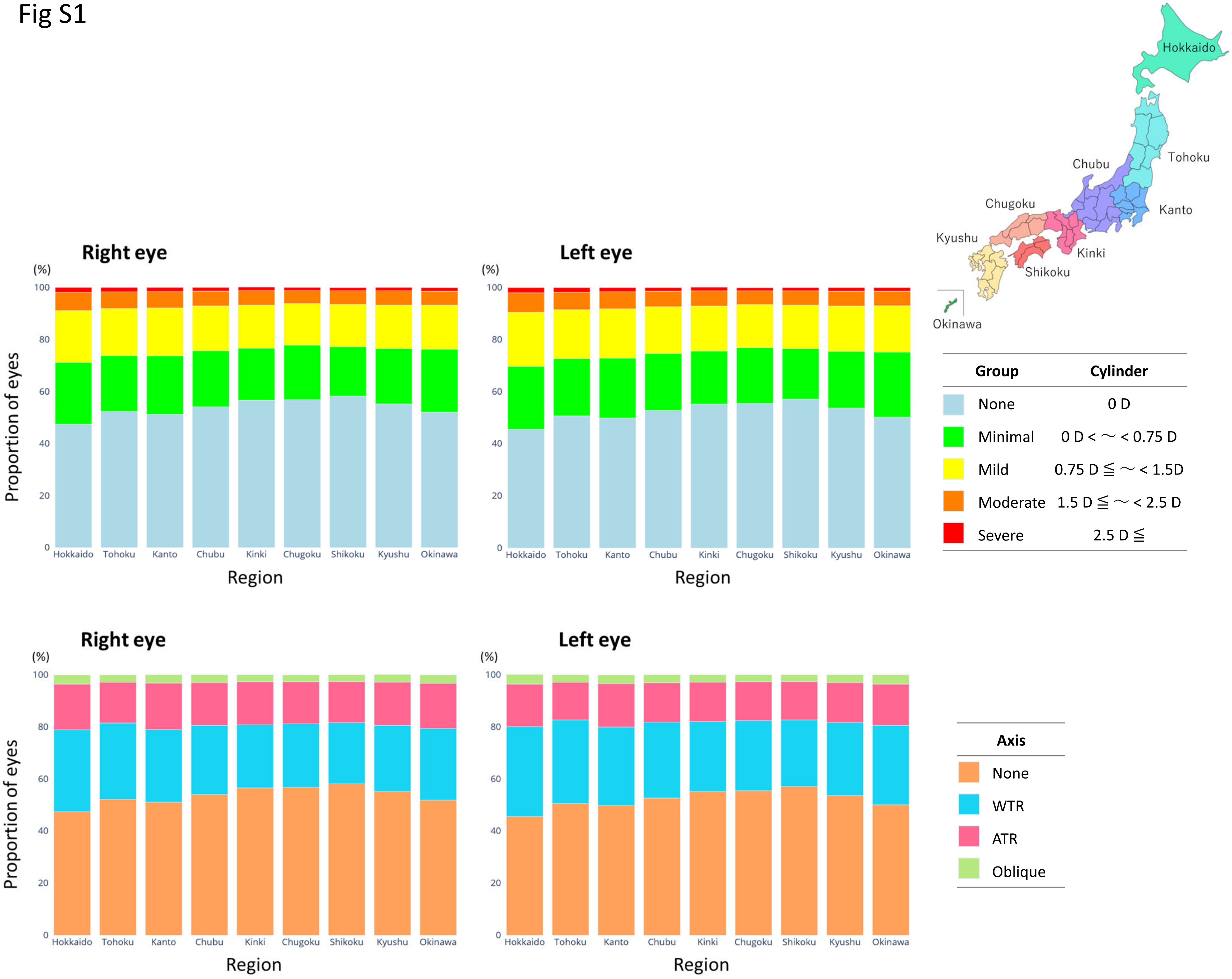

Hokkaido
Tohoku
Chubu
Kanto
Chugoku
Kinki
Kyushu
Shikoku
Okinawa
Right eye
Left eye
(%)
100
80
60
40
20
0
Proportion of eyes
Hokkaido Tohoku Kanto Chubu Kinki Chugoku Shikoku Kyushu Okinawa
Region
Group Cylinder
None 0 D
Minimal 0 D < ～ < 0.75 D
Mild 0.75 D ≦ ～ < 1.5D
Moderate 1.5 D ≦ ～ < 2.5 D
Severe 2.5 D ≦
Axis
None
WTR
ATR
Oblique

Supplementary Figure S1. Regional variation in cylinder power and astigmatic axis types across the nine geographic regions of Japan.

Upper panels: Stacked bar charts show the distributions of astigmatism severity (none, minimal, mild, moderate, and severe) for the Hokkaido, Tohoku, Kanto, Chubu, Kansai, Chugoku, Shikoku, Kyushu, and Okinawa regions. The regional differences were small but noticeable.

Lower panels: The proportions of the axis categories (WTR, ATR, oblique, and none) are provided for each region. Some differences in the distributions of the axis types were observed although the regional variations were modest.
