# Supplementary material for: Epidemiology of Astigmatism in Japan: Analysis of More Than 9,000,000 Spectacle Prescriptions
Source: Invest Ophthalmol Vis Sci. 2026 Apr 28;67(4):67. doi: 10.1167/iovs.67.4.67 (PMC13150933; doi:10.1167/iovs.67.4.67)
Supplement: Supplement 2 [file iovs-67-4-67_s002.pdf]

Supplementary Table S1.

Sample size and distribution of participants by geographic region, age group, and sex (n, row %, and column %)

	Female								Male								Row total
	6-19	20-29	30-39	40-49	50-59	60-69	70-79	80-89	6-19	20-29	30-39	40-49	50-59	60-69	70-79	80-89	
	27,907	38,259	29,659	31,885	22,480	7,260	2,239	469	23,614	28,493	26,906	31,095	21,466	7,423	1,949	208	
**Hokkaido**	9.3%	12.7%	9.8%	10.6%	7.5%	2.4%	0.7%	0.2%	7.8%	9.5%	8.9%	10.3%	7.1%	2.5%	0.6%	0.1%	301,312
	3.2%	3.7%	3.8%	3.6%	3.1%	2.8%	2.6%	2.3%	3.0%	3.3%	3.3%	3.3%	2.9%	2.6%	2.2%	1.5%	
	60,950	69,867	56,324	58,020	41,840	14,929	4,290	996	50,664	53,963	52,831	56,232	40,040	15,345	3,739	536	
**Tohoku**	10.5%	12.0%	9.7%	10.0%	7.2%	2.6%	0.7%	0.2%	8.7%	9.3%	9.1%	9.7%	6.9%	2.6%	0.6%	0.1%	580,566
	7.0%	6.7%	7.2%	6.5%	5.8%	5.8%	4.9%	5.0%	6.5%	6.2%	6.6%	5.9%	5.4%	5.4%	4.2%	3.9%	
	349,929	429,493	323,766	373,766	326,493	113,401	39,302	9,472	323,865	378,217	353,845	426,337	356,549	135,404	43,747	6,702	
**Kanto**	8.8%	10.8%	8.1%	9.4%	8.2%	2.8%	1.0%	0.2%	8.1%	9.5%	8.9%	10.7%	8.9%	3.4%	1.1%	0.2%	3,990,288
	40.3%	41.3%	41.2%	41.9%	44.9%	44.0%	44.9%	47.1%	41.6%	43.8%	43.9%	44.9%	48.1%	47.6%	48.9%	48.6%	
	134,571	160,688	118,677	131,714	100,832	34,490	11,359	2,452	117,397	130,348	121,051	135,858	97,843	35,636	10,354	1,691	
**Chubu**	10.0%	11.9%	8.8%	9.8%	7.5%	2.6%	0.8%	0.2%	8.7%	9.7%	9.0%	10.1%	7.3%	2.6%	0.8%	0.1%	1,344,961
	15.5%	15.4%	15.1%	14.8%	13.9%	13.4%	13.0%	12.2%	15.1%	15.1%	15.0%	14.3%	13.2%	12.5%	11.6%	12.3%	
	145,718	172,515	123,811	145,329	124,016	44,218	15,816	3,525	135,295	143,104	126,156	153,574	123,782	49,263	16,630	2,564	
**Kansai**	9.6%	11.3%	8.1%	9.5%	8.1%	2.9%	1.0%	0.2%	8.9%	9.4%	8.3%	10.1%	8.1%	3.2%	1.1%	0.2%	1,525,316
	16.8%	16.6%	15.8%	16.3%	17.1%	17.2%	18.1%	17.5%	17.4%	16.6%	15.6%	16.2%	16.7%	17.3%	18.6%	18.6%	
	46,849	54,389	39,166	45,933	34,133	12,420	4,615	1,111	40,407	42,016	38,157	44,242	31,142	12,298	3,987	717	
**Chugoku**	10.4%	12.0%	8.7%	10.2%	7.6%	2.8%	1.0%	0.2%	8.9%	9.3%	8.4%	9.8%	6.9%	2.7%	0.9%	0.2%	451,582
	5.4%	5.2%	5.0%	5.1%	4.7%	4.8%	5.3%	5.5%	5.2%	4.9%	4.7%	4.7%	4.2%	4.3%	4.5%	5.2%	
	23,985	23,209	19,731	23,805	17,572	6,876	2,386	527	20,417	18,402	18,658	22,968	15,509	6,428	2,175	343	
**Shikoku**	10.8%	10.4%	8.8%	10.7%	7.9%	3.1%	1.1%	0.2%	9.2%	8.3%	8.4%	10.3%	7.0%	2.9%	1.0%	0.2%	222,991
	2.8%	2.2%	2.5%	2.7%	2.4%	2.7%	2.7%	2.6%	2.6%	2.1%	2.3%	2.4%	2.1%	2.3%	2.4%	2.5%	
	70,397	81,176	65,438	72,308	53,178	21,619	6,934	1,436	59,377	61,848	60,980	71,151	49,515	20,919	6,398	936	
**Kyushu**	10.0%	11.5%	9.3%	10.3%	7.6%	3.1%	1.0%	0.2%	8.4%	8.8%	8.7%	10.1%	7.0%	3.0%	0.9%	0.1%	703,610
	8.1%	7.8%	8.3%	8.1%	7.3%	8.4%	7.9%	7.1%	7.6%	7.2%	7.6%	7.5%	6.7%	7.4%	7.2%	6.8%	
	8,683	10,684	9,130	9,209	6,169	2,241	557	122	6,839	7,543	7,537	7,821	5,471	1,873	402	86	
**Okinawa**	10.3%	12.7%	10.8%	10.9%	7.3%	2.7%	0.7%	0.1%	8.1%	8.9%	8.9%	9.3%	6.5%	2.2%	0.5%	0.1%	84,367
	1.0%	1.0%	1.2%	1.0%	0.8%	0.9%	0.6%	0.6%	0.9%	0.9%	0.9%	0.8%	0.7%	0.7%	0.4%	0.6%	
**Column total**	868,989	1,040,280	785,702	891,969	726,713	257,454	87,498	20,110	777,875	863,934	806,121	949,278	741,317	284,589	89,381	13,783	9,204,993

Data are presented as n, row %, and column % within each sex and age group. Age groups are categorized in 10-year intervals (6–10, 20–29, etc.).

Row % indicates the distribution of participants across age groups within each geographic region. Column % indicates the proportion of participants from each region within each age group and sex. Row total indicates the total number of individuals in each region. Column total indicates the total number of individuals by age group and sex.
