# Supplementary material for: Epidemiology of Astigmatism in Japan: Analysis of More Than 9,000,000 Spectacle Prescriptions
Source: Invest Ophthalmol Vis Sci. 2026 Apr 28;67(4):67. doi: 10.1167/iovs.67.4.67 (PMC13150933; doi:10.1167/iovs.67.4.67)
Supplement: Supplement 3 [file iovs-67-4-67_s003.pdf]

Supplementary Table S2.
Relative importance of age, sex, and region in multivariable analyses

Multivariable analyses	Measure	Eye	Age	Sex	Region
Multiple linear regression	Partial coefficient of determination (Partial R^2)	Right	0.0301	0.0077	0.0017
		Left	0.0205	0.0060	0.0017
	Relative importance*	Right	58.81%	29.34%	11.84%
		Left	55.38%	29.80%	14.81%
Multinomial logistic regression	Partial pseudo–coefficient of determination (Pseudo-R^2)	Right	0.0671	0.0041	0.0014
		Left	0.0680	0.0040	0.0014
	Relative importance**	Right	92.57%	6.71%	0.71%
		Left	92.47%	6.71%	0.82%

Values represent the relative contribution of each variable to the model.

* Calculated based on standardized regression coefficients.

** Calculated based on permutation importance.
